# Contact Irritant Responses of *Aedes aegypti* Using Sublethal Concentration and Focal Application of Pyrethroid Chemicals

**DOI:** 10.1371/journal.pntd.0002074

**Published:** 2013-02-28

**Authors:** Hortance Manda, Pankhil Shah, Suppaluck Polsomboon, Theeraphap Chareonviriyaphap, Fanny Castro-Llanos, Amy Morrison, Roxanne G. Burrus, John P. Grieco, Nicole L. Achee

**Affiliations:** 1 Uniformed Services University of the Health Sciences, Department Preventive Medicine and Biometrics, Bethesda, Maryland, United States of America; 2 Department of Entomology, Faculty of Agriculture, Kasetsart University, Bangkok, Thailand; 3 Naval Medical Research No. 6 (NAMRU-6), Lima, Peru; 4 Department of Entomology, University of California, Davis, Davis, California, United States of America; Mahidol University, Thailand

## Abstract

**Background:**

Previous studies have demonstrated contact irritant and spatial repellent behaviors in *Aedes aegypti* following exposure to sublethal concentrations of chemicals. These sublethal actions are currently being evaluated in the development of a push-pull strategy for *Ae. aegypti* control. This study reports on mosquito escape responses after exposure to candidate chemicals for a contact irritant focused push-pull strategy using varying concentrations and focal application.

**Methods:**

Contact irritancy (escape) behavior, knockdown and 24 hour mortality rates were quantified in populations of female *Ae. aegypti* under laboratory conditions and validated in the field (Thailand and Peru) using experimental huts. Evaluations were conducted using varying concentrations and treatment surface area coverage (SAC) of three pyrethroid insecticides: alphacypermethrin, lambacyhalothrin and deltamethrin.

**Results:**

Under laboratory conditions, exposure of *Ae. aegypti* to alphacypermethrin using the standard field application rate (FAR) resulted in escape responses at 25% and 50% SAC that were comparable with escape responses at 100% SAC. Significant escape responses were also observed at <100% SAC using ½FAR of all test compounds. In most trials, KD and 24 hour mortality rates were higher in mosquitoes that did not escape than in those that escaped. In Thailand, field validation studies indicated an early time of exit (by four hours) and 40% increase in escape using ½FAR of alphacypermethrin at 75% SAC compared to a matched chemical-free control. In Peru, however, the maximum increase in *Ae. aegypti* escape from alphacypermethrin-treated huts was 11%.

**Conclusions/Significance:**

Results presented here suggest a potential role for sublethal and focal application of contact irritant chemicals in an *Ae. aegypti* push-pull strategy to reduce human–vector contact inside treated homes. However, the impact of an increase in escape response on dengue virus transmission is currently unknown and will depend on rate of biting on human hosts prior to house exiting.

## Author Summary

Dengue virus is spread by *Aedes aegypti*, a mosquito that prefers to feed on humans. Chemicals used at toxic levels is currently the only confirmed effective strategy for dengue vector control, but insecticide resistance issues are threatening this approach. Other chemical actions that break vector-human contact exist: contact irritant effects, causing escape from homes and spatial repellent effects, preventing house entry. These actions are being evaluated in the development of a push-pull strategy to target indoor resting sites (using contact irritants) and/or portals of house entry (using spatial repellents) at sublethal concentrations to push the vector away from the treated structure and associated human hosts. When combined with an attractant trap placed outdoors to pull the irritated/repelled vectors from the peridomestic environment, the integrated stimuli form a push-pull system that can be used to further enhance disruption of human-vector contact. Here we report on the quantification of contact irritancy (escape) behavior, knockdown and 24 hour mortality rates in populations of *Ae. aegypti* under both laboratory and field conditions. Evaluations were carried out against three pyrethroid insecticides. Findings indicate an increase in escape response using sublethal concentrations and focal application suggesting a potential role for contact irritants in a push-pull strategy.

## Introduction

Dengue, transmitted primarily by *Aedes aegypti* mosquitoes, is the most important mosquito-borne viral disease affecting humans worldwide [1]. It is caused by four serotypes that produce a spectrum of clinical illness ranging from unapparent or mild disease, to an influenza-like illness, to a fatal shock syndrome.

Due to the current lack of a licensed vaccine, dengue prevention is limited to vector control. *Aedes aegypti* control programs are based on two main targets: (1) the immature stages (egg, larvae, and pupae) through environmental management (source reduction), larvicides and/or biological control; and (2) the adult stage using space or residual sprays of chemical insecticides and more recently insecticide treated materials [2]. *Aedes aegypti* has strong associations with human habitations, living and breeding very near or inside human dwellings [3]–[5]. This extensive use of the human indoor environment poses challenges to traditional adult control methods and as well as in devising new or improved methods to sufficiently reduce disease transmission risk [6]. Pyrethroids have commonly been employed in dengue endemic countries such as Thailand and Peru for peridomestic and/or indoor residual/space-spraying to reduce adult mosquito populations [7], [8]. However, despite the fact that selective use of pyrethroids and other residual insecticides applied indoors have successfully controlled *Ae. aegypti* and dengue [9]–[13], outdoor and peridomestic space-spraying alone has often failed to achieve any meaningful control of indoor adult *Ae. aegypti* populations. This is because the chemical fails to reach the preferred resting sites inside homes [14], [15] and indoor residual or space-spraying can be resource limited and hampered by poor public perception that limits indoor access [16] therefore reducing treatment coverage. However, the high affinity of *Ae. aegypti* for the human indoor environment also provides unique opportunities for innovative approaches to control the adult vector [16].

During dengue outbreaks, the methods of choice for emergency interventions remain outdoor ultra-low-volume (ULV) application of insecticides and/or indoor thermal fogging [17], [18]. While effective, these interventions usually follow onset of epidemics, are difficult to implement in urban environments where *Ae. aegypti* is most common, and more importantly very difficult to sustain [16]. For these and other reasons such as insecticide resistance [7], [8], there is a need for proactive measures that have potential to prevent virus transmission and avert dengue epidemics as well as reduce selection pressure for insecticide resistance.

Research has shown that the impact of public health insecticides on vector populations is much more complex than simple toxic actions (i.e., direct mortality following exposure). There are other chemical actions that exist to break human-vector contact [19]–[28]. Such actions include among others, contact irritant effects, causing an escape response from homes, and initiating a spatial repellent or deterrent effect, thereby preventing house entry [26], [28], [29]. Both contact irritant and spatial repellent behaviors have been demonstrated in vector populations using sublethal chemical concentrations under both laboratory and field environments [26], [28]. The importance of these findings is accentuated as both organochlorine resistant and pyrethroid tolerant *Ae. aegypti* were included in the vector test populations, indicating that behavior-modifying actions exist even when mosquitoes are not susceptible to their toxic actions and as such, may play a role in insecticide resistance management. Current issues of insecticide resistance are creating a necessity for development of new strategies [30] and/or chemical products that act to prevent vector biting. Such approaches will require an understanding of the range of biological actions (independent from toxicity) elicited by vectors exposed to chemical tools to help drive such methods for disease control.

Both contact irritancy and spatial repellency are currently being evaluated in the development of a push-pull strategy for *Ae. aegypti* control. The goal of the push-pull strategy is to reduce the probability of human-vector contact and therefore dengue virus transmission. The approach is to target preferred indoor resting sites (using contact irritants) and house portals of entry (using spatial repellents) using minimum effective chemical concentration and treatment surface area coverage (SAC) to make these sites unsuitable, and thereby drive (push) the vector away from the treated structure and human hosts in a cost-effective manner. To further enhance the effect of a push response, an attractant trap is placed outdoors to pull the irritated/repelled vectors from the peridomestic environment thereby disrupting human-vector contact in the peridomestic environment. However, a component to system success based on a contact irritant push element is the quantification of a contact irritant response - movement of *Ae. aegypti* test populations away from a chemical source following tarsal contact–when less than 100% of a surface is treated (i.e. focal application). Target application could prove cost-effective; however, untreated sites inside homes may serve as refuge locations for exposed vectors thereby diminishing efficacy of the approach.

Our previous study quantified *Ae. aegypti* resting behavior under laboratory conditions following exposure to chemical-treated surfaces at various treatment coverage area (100%, 75%, 50% and 25%) to define the effects of “safe sites” (untreated surfaces) on irritancy behavior [31]. Results indicated that when preferred *Ae. aegypti* resting sites were treated with irritant chemicals, even at a treatment coverage of 25%, test populations did not simply move to untreated areas but became agitated, using increased flight as a proxy indicator. It is this contact irritant response that may result in an escape behavior and could be exploited as one type of push component in a push-pull strategy.

The objectives of the current study were to quantify contact irritancy (escape) response, knockdown, and 24 hour mortality rates in populations of female *Ae. aegypti*. Evaluations were carried out at varying chemical concentrations and treatment surface area coverage using three standard pyrethroid insecticides: alphacypermethrin, lambdacyhalothrin and deltamethrin. We present findings from the laboratory and two field sites (Thailand and Peru) where validation studies using experimental huts were conducted.

## Methods

### Mosquitoes

#### Laboratory

*Aedes aegypti* test populations (F_2_-F_5_ generations) from Iquitos, Peru (PERU strain) were used for all laboratory experiments. Larvae were reared from eggs shipped to the Uniformed Services University of the Health Sciences (USUHS), Bethesda, USA from the Naval Medical Research Unit No. 6 (NAMRU-6) Iquitos Entomology Laboratory, Iquitos, Peru (INRENA authorization number 128-2007-INRENA-IFFS-DCB, permit number 000703-AG-DGFFS), following previously described protocols [32]. Females (4–7 days old) were provided with sugar pads saturated in a 10% sucrose solution until 24 hours prior to the day of testing. The USUHS colonies were maintained using a membrane blood-feeding system until the F_5_ generation at which time the colony was refreshed with F_1_ field material to control for behavioral comparability between laboratory and field populations.

#### Field

*Aedes aegypti* were collected as immatures from experimental hut locations: Pu Teuy Village, Kanchanaburi Province, Thailand (THAI strain); and Iquitos, Peru (PERU strain). Larval populations were reared to adults at respective field insectaries using site-specific established protocols and colonized until the F5 generation. *Aedes aegypti* from Pu Teuy Thailand has been described as pyrethroid tolerant [27], [33]; while a recent survey from neighborhood sites in Iquitos, Peru have reported *Ae. aegypti* populations susceptible to pyrethroids (Vasquez La Torre, personal communication). As with laboratory studies, mosquito test populations were maintained with sugar pads saturated in a 10% sucrose solution until 24 hours prior to day of testing. Test cohorts (100 per hut) were marked with a unique color fluorescent dust (BioQuip Products, Inc., Gardena CA) to facilitate visual observation of mosquitoes during experimental hut trials. The use of a unique marking color per hut also allowed to determine the total number of mosquitoes that were present inside each hut during each experimental day, and thus defining proportion escape using a known denominator.

### Test compounds

Chemicals evaluated were chosen based on current World Health Organization Pesticide Evaluation Scheme (WHOPES) recommendations and/or historical use in vector control programs [34]. Compounds were acquired as neat grade material purchased from Sigma-Aldrich (St. Louis, MO): alphacypermethrin (CAS 67375-30-8), lambdacyhalothrin (CAS 91465-08-6), and deltamethrin (CAS 52918-63-5). Test concentrations evaluated included WHO recommended field application rates (FAR) and ½FAR; where FAR = 7.2 nmol/cm^2^ (0.03 g/m^2^) for alphacypermethrin and lambdacyhalothrin, and 4.9 nmol/cm^2^ (0.025 g/m^2^) for deltamethrin.

### Laboratory evaluations

#### Assay device

The previously described box assay was used for all laboratory evaluations [31]. Briefly, the assay is composed of metal and Plexiglas chambers (30 cm^3^) that are joined together using draw latches. The metal test chamber is fitted with textile panels (either chemical-free or treated) and has a funneled exit portal through which mosquitoes can escape into the Plexiglas receiving chamber. The metal test chamber is covered with a Plexiglas lid to facilitate viewing of mosquito behavior during testing, as well as, a removable tinted lid that can be added to maintain darkness throughout the test procedure as desired. A full description of the assay along with accompanying standard operating procedures can be found at www.usuhs.mil/pmb/gsvc.

#### Contact irritant assay

Laboratory studies were conducted using both black and white cotton fabric (Natural Charm 43/44″ wide 100% cotton 68×68 D/R-black and white, Bruce Variety, Bethesda, MD, USA) based on results from previous *Ae. aegypti* resting preference evaluations [31]. All fabric pieces were treated approximately 30 min prior to initiating the first assay replicate and allowed to air-dry for at least 15 min prior to inserting it into the test chambers. New fabric panels were prepared daily. Treated and chemical-free cotton were fitted into the metal chamber at 100% dark (D), 100% light (L), and 75%∶25%, 50%∶50%, and 25%∶75% D∶L surface area coverage (SAC) ratios [31]. Only dark material strips were treated with chemical solutions except during 100%L evaluations. Fabric panels were attached to assay walls using magnets. Groups of 20 female mosquitoes were introduced into the metal chamber and allowed to rest for 30 sec after which time the funnel gate linking the metal and Plexiglas chambers was opened to allow mosquito escape during a 10 min sampling period. The following data was then collected: 1) the number of *Ae. aegypti* in the Plexiglas chamber (i.e., escaping); 2) the number of mosquitoes within the metal chamber (mosquitoes that did not escape) and; 3) knockdown (KD) (defined as mosquitoes lying on their side or back that are unable to right themselves) in both clear and metal chambers. Mosquitoes were immediately removed from the assay using vacuum power hand aspirators and placed into containers labeled according to the respective chamber and maintained at 28°C and 80% RH to monitor 24 hour mortality. A total of 6 replicates were performed for each chemical concentration and D∶L coverage. A matched control assay fitted with acetone-only treated fabric at the same D∶L coverage was conducted simultaneously for each replicate. All testing was performed under controlled temperature (28–30°C) and relative humidity (50–60%) conditions. Box assays were cleaned with a 10% bleach solution at the end of each testing day to control for residual chemical and allowed to air-dry overnight before reuse.

### Field evaluations

#### Experimental huts

**Figure 1:**
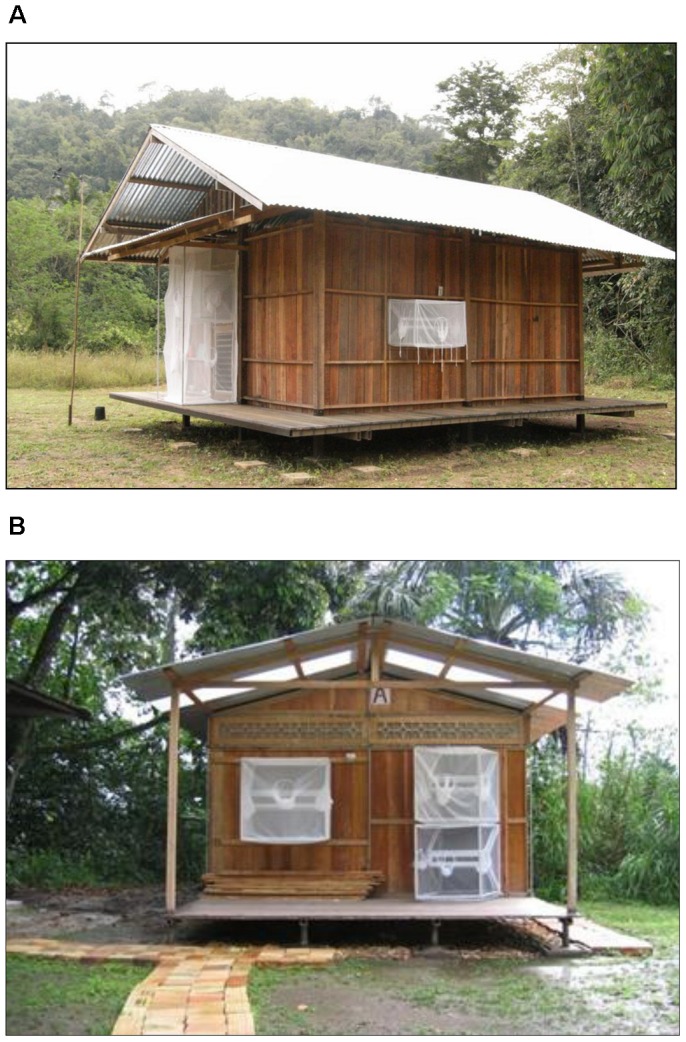
Experimental huts fitted with window and door interception traps. (A) Thailand, (B) Peru.

Trials were conducted in both Thailand and Peru using experimental huts fitted with window and door interception traps. Three (Thailand) and five (Peru) portable huts were constructed using dimensions and materials that mimic indigenous homes at each site (Figure 1). Details of the experimental hut and intercept trap design in Thailand have been previously described [35]. In Peru, the huts measured 4 m wide×6 m long×6 m height. Each hut had two windows (96 cm wide×96 cm height) and two doors (1 m wide×2 m height); one window and one door each at the front and the back, which were fitted with interception traps. At both field sites, the huts were positioned on raised platforms 30 cm above ground. The support columns for the platform sit atop cement ant traps to prevent predation on indoor KD mosquitoes. Floors were covered with white plastic sheeting to facilitate the detection of KD responses. Huts at both locations contained metal frame panels with wire-mesh backing positioned along the interior walls. Treated fabric panels were fixed to these metal panels using magnets, thereby avoiding chemical contact with the wall surface and potential contamination during subsequent chemical testing or treatment concentration. Environmental parameters of indoor/outdoor temperature and relative humidity were recorded using HOBO data loggers (series H08-004-02 and H21-001, respectively). HOBO U30/NRC Data Logging Weather Stations (Onset Computer Corporation Cape Cod, MA, USA) were used at both sites and were positioned centrally to the experimental huts.

#### Escape trials

Black and white cotton (Consorcio la Parcela Products, Ate, Lima, Peru; size 39.4×0.8/inch); and green and white polyester (BioQuip Products, Rancho Dominguez, CA, USA; mesh size 24×20/inch) fabric panels were used in Peru and Thailand, respectively. Experimental design for measuring mosquito escape behavior followed previously established protocols [26]. Briefly, fabric panel strips were measured to cover a specific surface area coverage (SAC) of interior hut walls. Chemical solutions were prepared using either acetone (in Thailand) or absolute ethanol (in Peru) to test concentration and applied to fabric panels using a pipette. Additional fabric panels were treated with solvent only, to serve as matched chemical-free controls. Panels were treated no more than 48 hours prior to initiating the first replicate of a trial. Following treatment, the fabric was wrapped in aluminum foil and placed under cold storage (4°C) for transport to the field. The day before testing, chemical-treated and chemical-free fabric panels were affixed onto the metal frames inside each hut at 100% D, 75%∶25%, 50%∶50%, and 25%∶75% D∶L ratios where only dark fabric was chemical-treated. A matched control hut with similar SAC was performed simultaneously for each D∶L trial using solvent-only treated fabric.

Interception traps were affixed on the outside of doors and windows to capture exiting mosquitoes. Three to seven day-old marked female *Ae. aegypti* (100 per hut) were released inside each hut at 05.30 Hrs each experimental day. An additional container holding 25 marked mosquitoes was positioned in the center of each hut to serve as an internal control for monitoring KD due to indoor environmental conditions. During testing, one person was positioned indoors underneath an untreated bed net to generate host cues and monitor hourly KD. From 06.00 Hrs–18.00 Hrs at 20 min (Thailand) and 30 min (Peru) intervals, collector pairs located outside each hut removed mosquitoes within interception traps. Captured mosquitoes were placed into individual holding cups labeled by hut, trap number and time. At the top of each hour, all KD mosquitoes on the floor were collected and placed into holding cups labeled by hut and time. Both escape and KD mosquito samples were provided with cotton pads soaked in 10% sugar solution and held overnight in field insectaries to monitor 24 hour mortality. Four (Thailand) or five (Peru) replicates (i.e. experimental days) were performed for each experimental trial. Outdoor collector pairs rotated between huts every sampling period and indoor hosts at mid-day during an experimental day to control for individual bias. At the end of each experimental day, all mosquitoes that remained inside the huts were collected using backpack (Thailand) and Prokopack (Peru) [36] aspiration. The following treatments were tested in Thailand: alphacypermethrin at FAR and ½FAR at 75% D∶L SAC; and in Peru: alphacypermethrin at FAR and ½FAR at 100%, 75%, 50%, and 25% D∶L SAC.

### Data analysis

#### Laboratory evaluations

All analyses were performed using SAS v. 9.2 software [37]. Proportional data were subjected to arcsine square root transformation before statistical analysis. Output tables represent back-transformed values. Mean percentage escaping (or corrected percentage escaping) was obtained from the number escaping in the treatment over the number released after correcting for the number escaping in the control and KD in the metal test chambers). Knockdown and 24 hour mortality rates of populations that escaped and those that did not escape were corrected based on measurements from populations in control assays using Abbott's formula [38]. For each chemical dose and SAC ratio trial, the difference between the number escaping from treated and control test chambers; and the difference between KD or dead in mosquitoes that escaped and those that did not escape were analyzed using the Wilcoxon 2-sample test as previously described [26], [28], [39]. Variations in the percentage of mosquitoes escaping, KD and dead after 24 hours per trial were analyzed for each chemical by 2-way analysis of variance (ANOVA) with 2 main factors (treatment concentration and surface area coverage). For those chemicals that showed significant variations, an additional one-way ANOVA was conducted to examine the main factors (treatment concentration or SAC ratio), independently, on percentage escaping, KD and mortality. Means were separated using t-test and Sheffe's test (α = 0.05).

#### Field evaluations

The mean daily percentages of mosquitoes that escaped, indoor KD and 24 hour mortality rates from those collected inside the huts (mosquitoes that did not escape) and those that escaped were compared between treatment and control huts using one-way ANOVA with means separated using Student Newman Keuls (SNK) tests (α = 0.05). The percentage of mosquitoes that escaped was compared between huts at three time periods: 06.00–10.00 Hrs, 11.00–14.00 Hrs and 15.00–18.00 Hrs corresponding to expected morning, mid-day and afternoon *Ae. aegypti* resting behavior patterns (Castro et al. unpublished data). Percentage escape was calculated using the number of mosquitoes that escaped over the number of mosquitoes that were available to escape (total number of mosquitoes recaptured from the hut each day minus number knockdown within the hut).

## Results

### Laboratory evaluations

#### Escape responses

The mean number of female *Ae. aegypti* escaping in the treatment and matched control assays, with corrected percentage escaping for all trials is presented in Tables 1–3. In general, escape was higher in treatment assays as compared to matched controls. Most importantly, significant escape response was observed at ½FAR and/or at SAC less than 100% for each pyrethroid evaluated (Tables 1–3). Against alphacypermethrin treatment, corrected percent escape ranged from 8–49% with highest responses observed at FAR for all SAC evaluated (Table 1). Percent escape was comparable among all treatment coverages at the ½FAR dose (Table 1). With lambdacyhalothrin and deltamethrin treatments, corrected percent escape ranged from 1–59% (Table 2) and from −1–40% (Table 3), respectively. At each treatment concentration, escape response was highest using 100%L SAC, while for each treatment coverage (except 100%L), escape response was comparable among doses (Tables 2 and 3).

**Table 1:** Escape responses of *Aedes aegypti* 1 to different concentrations and coverage of alphacypermethrin under laboratory conditions.

				No. escaping (mean^2^ ± SE)				
Alphacypermethrin concentration (nmoles/cm^2^)	SAC^3^ (%)	No. of mosquitoes Treatment	No. of mosquitoes Control	Treated	Control	% escaping^4^ (mean ± SE)	*P* 5	*P* 6
½FAR^7^ (3.6)	25	119	119	0.0±0.0	0.2±0.2	8±4^a^	0.06	<0.01
	50	118	119	0.2±0.2	0.5±0.3	8±4^a^	0.02	<0.01
	75	118	118	0.2±0.2	0.0±0.0	8±5^a^	0.06	0.30
	100D	118	116	0.2±0.2	0.0±0.0	20±4^a^	<0.01	<0.01
	100L	117	121	1.8±0.6	0.5±0.5	24±6^a^	<0.01	0.36
	*P* 8			-	-	0.05		
FAR (7.2)	25	123	120	0.3±0.2	0.0±0.0	35±6^ab^	<0.01	
	50	118	120	0.5±0.2	0.3±0.2	35±5^ab^	<0.01	
	75	118	125	0.7±0.3	0.2±0.2	14±4^b^	<0.01	
	100D	119	118	0.8±0.7	0.2±0.2	49±6^a^	<0.01	
	100L	108	116	6.5±0.6	5.2±1.0	36±11^ab^	0.01	
	*P*			-	-	0.01		

1 Four to seven day-old females, non-blood-fed, 24 hour sugar starved (PERU).

2 For each trial (n = 6 replicates).

3 Surface area coverage (SAC) of treated material.

4 For each trial percentage escaping after correction based on escape in the control and knockdown in the metal test chambers using Abbott's formula. Means in the same column followed by the same letter were not significantly different. Multiple comparisons of means were done using Scheffe's test (α = 0.05).

5 P values are from Wilcoxon 2-sample test for difference between the number escaping in a chemical treatment chamber and an acetone treatment (control) chamber.

6 P values are from t-test examining the effect of treatment concentrations on corrected percentage escaping at each treatment coverage.

7 WHO recommended field application rate (FAR) = 7.2 nm/cm^2^ or 0.03 g/m^2^.

8 P values are from one-way ANOVA examining the effect of treatment coverage on corrected percentage escaping at each treatment concentration.

**Table 2:** Escape responses of *Aedes aegypti* 1 to different concentrations and coverage of lambdacyhalothrin under laboratory conditions.

				No. escaping (mean^2^ ± SE)				
Lambdacyhalothrin concentration (nmoles/cm^2^)	SAC^3^ (%)	No. of mosquitoes) Treatment	No. of mosquitoes) Control	Treated	Control	% escaping^4^ (mean ± SE)	*P* 5	*P* 6
½FAR^7^ (3.6)	25	119	122	2.7±0.6	0.8±0.5	12±3^a^	0.03	0.99
	50	120	122	4.0±0.7	2.3±0.7	9±9^a^	0.16	0.23
	75	116	114	6.2±1.0	3.3±0.4	15±11^a^	0.08	0.50
	100D	115	114	0.5±0.3	0.0±0.0	1±4^a^	0.45	0.22
	100L	118	122	6.2±1.3	0.2±0.2	38±5^a^	<0.01	0.03
	*P* 8			-	-	0.06		
FAR (7.2)	25	122	122	3.0±0.8	1.0±0.5	13±5^b^	0.08	
	50	116	122	3.2±0.7	1.0±0.4	21±2^b^	0.01	
	75	114	117	4.3±1.0	0.8±0.5	23±7^b^	<0.01	
	100D	116	115	3.0±0.9	0.3±0.2	13±8^b^	0.04	
	100L	119	116	11.2±1.8	1.0±0.4	59±9^a^	<0.01	
	*P*			-	-	<0.01		

1 Four to seven day-old females, non-blood-fed, 24 hour sugar starved (PERU).

2 For each trial (n = 6 replicates).

3 Surface area coverage (SAC) of treated material.

4 For each trial percentage escaping after correction based on escape in the control and knockdown in the metal test chambers using Abbott's formula. Means in the same column followed by the same letter were not significantly different. Multiple comparisons of means were done using Scheffe's test (α = 0.05).

5 P values are from Wilcoxon 2-sample test for difference between the number escaping in a chemical treatment chamber and an acetone treatment (control) chamber.

6 P values are from t-test examining the effect of treatment concentrations on corrected percentage escaping at each treatment coverage.

7 WHO recommended field application rate (FAR) = 7.2 nm/cm^2^ or 0.03 g/m^2^.

8 P values are from one-way ANOVA examining the effect of treatment coverage on corrected percentage escaping at each treatment concentration.

**Table 3:** Escape responses of *Aedes aegypti* 1 to different concentrations and coverage of deltamethrin under laboratory conditions.

				No. escaping (mean^2^ ± SE)				
Deltamethrin concentration (nmoles/cm^2^)	SAC^3^ (%)	No. of mosquitoes) Treatment	No. of mosquitoes) Control	Treated	Control	% escaping^4^ (mean^2^ ± SE)	*P* 5	*P* 6
½FAR^7^ (2.47)	25	118	115	0.3±0.2	0.0±0.0	−1±3^b^	0.45	0.30
	50	121	119	1.2±0.5	0.3±0.2	2±3^ab^	0.27	0.89
	75	114	127	4.5±0.7	1.7±0.8	24±9^a^	0.03	0.11
	100D	121	120	0.7±0.3	0.2±0.2	2±5^ab^	0.30	0.16
	100L	115	123	4.0±1.0	0.2±0.2	26±4^a^	<0.01	<0.01
	*P* 8			-	-	<0.01		
FAR (4.94)	25	121	119	1.2±0.5	0.0±0.0	6±5^b^	0.06	
	50	118	120	0.8±0.5	0.0±0.0	5±6^b^	0.45	
	75	121	114	2.0±0.5	0.0±0.0	7±6^b^	0.01	
	100D	122	118	2.7±0.5	0.0±0.0	11±5^b^	<0.01	
	100L	119	118	7.8±1.2	0.2±0.2	40±6^a^	<0.01	
	*P*			-	-	<0.01		

1 Four to seven day-old females, non-blood-fed, 24 hour sugar starved (PERU).

2 For each trial (n = 6 replicates).

3 Surface area coverage (SAC) of treated material.

4 For each trial percentage escaping after correction based on escape in the control and knockdown in the metal test chambers using Abbott's formula. Means in the same column followed by the same letter were not significantly different. Multiple comparisons of means were done using Scheffe's test (α = 0.05).

5 P values are from Wilcoxon 2-sample test for difference between the number escaping in a chemical treatment chamber and an acetone treatment (control) chamber.

6 P values are from t-test examining the effect of treatment concentrations on corrected percentage escaping at each treatment coverage.

7 WHO recommended field application rate (FAR) = 4.9 nm/cm^2^ or 0.025 g/m^2^.

8 P values are from one-way ANOVA examining the effect of treatment coverage on corrected percentage escaping at each treatment concentration.

#### Knockdown and 24 hour mortality

In most trials, KD and 24 hour mortality rates were higher in *Ae. aegypti* that did not escape than in those that escaped (Table S1 and Table 4, respectively); however, there was minimal KD observed overall in test populations that escaped with rates ranging from 0–25% in all test chemicals. Highest KD was observed using alphacypermethrin at 100%D SAC and FAR (Table S1). No KD was observed in mosquitoes that escaped against lambdacyhalothrin or deltamethrin (Table S1). For mosquitoes that did not escape, percentage KD ranged from 0–48%, 0–15% and 0–1% using alphacypermethrin, lambdacyhalothrin and deltamethrin, respectively, with highest rates occurring in alphacypermethrin trials using FAR (Table S1). Most importantly, KD observed in both mosquito cohorts that escaped and those that did not were comparable between 100% and 25% SAC for each test concentration and chemical evaluated.

**Table 4:** Percentage mortality *Ae. aegypti* 1 that escaped and those that did not escape under laboratory conditions.

Chemicals	SAC^3^ (%)	Mean^2^ ± SE Percentage mortality					
		Escaped			Did not escape		
		½FAR^4^	FAR	*P* 5	½FAR	FAR	*P*
Alphacypermethrin	25	NA^7^	0±0^c^	-	49±15^a^	84±7^a^	0.04
	50	0±0^a^	0±0^c^	-	48±14^a^	73±8^a^	0.14
	75	0^a^	0±0^c^	-	45±11^a^	60±10^a^	0.32
	100D	100^a^	100±0^a^	-	47±10^a^	69±10^a^	0.14
	100L	18±13^a^	45±9^b^	0.06	54±7^a^	52±8^a^	0.84
	*P* 6	0.07	<0.01		0.98	0.11	
Lambdacyhalothrin	25	0±0^a^	10±10^a^	0.29	0.5±0.3^b^	20±7^a^	<0.01
	50	0±0^a^	5±5^a^	0.34	14±3^a^	28±7^a^	0.12
	75	8±8^a^	17±9^a^	0.37	8±4^ab^	17±4^a^	0.09
	100D	25±25^a^	0±0^a^	0.42	13±2^a^	17±7^a^	0.75
	100L	6±4^a^	10±6^a^	0.55	13±4^a^	25±5^a^	0.07
	*P*	0.27	0.67		<0.01	0.58	
Deltamethrin	25	0±0^a^	0±0^a^	-	8±4^ab^	5±3^a^	0.49
	50	0±0^a^	0±0^a^	-	1±1^b^	10±4^a^	0.02
	75	0±0^a^	7±7^a^	0.29	5±2^ab^	4±2^a^	0.88
	100D	0±0^a^	0±0^a^	-	7±3^ab^	10±4^a^	0.46
	100L	6±6^a^	2±2^a^	0.66	16±4^a^	7±3^a^	0.09
	*P*	0.68	0.64		0.02	0.36	

1 Four to seven day old females, non-blood-fed, 24 hour sugar starved (PERU).

2 For each trial (n = 6 replicates), percentage 24 h mortality is corrected for control using Abbot's formula. Means in the same column followed by the same letter were not significantly different. Multiple comparisons of means were done using Scheffe's test (α = 0.05).

3 Surface area coverage (SAC) of treated material.

4 WHO recommended field application rate (FAR) = 7.2 nm/cm^2^ or 0.03 g/m^2^ for alphacypermethrin and lambdacyhalothrin; and 4.9 nm/cm^2^ or 0.025 g/m^2^ for deltamethrin.

5 P values are from t-test examining the effect of treatment concentrations on corrected percentage 24 h mortality at each treatment coverage.

6 P values are from one-way ANOVA for difference in corrected percentage 24 h mortality between treatment coverage at each treatment concentration.

7 NA = Not available (no escapee).

In general, 24 hour mortality rates were highest in mosquitoes that did not escape compared to *Ae. aegypti* test populations that escaped the laboratory assay (Table 4). Mortality ranged from 0–100%, 0–25%, and 0–7% in mosquitoes that escaped and from 45–84%, 0.5–28%, and 1–16% in those that did not escape for alphacypermethrin, lambdacyhalothrin and deltamethrin, respectively. For alphacypermethrin, highest mortality was observed at FAR 100%D in mosquitoes that escaped and 25%D in those that did not escape, although the latter was statistically comparable among all SAC using both test concentrations (Table 4). Percentage mortality in mosquitoes that escaped was comparable among all SAC using each test concentration of lambdacyhalothrin and deltamethrin (Table 4). Trials using ½FAR lambdacyhalothrin and deltamethrin indicated percentage mortality in mosquitoes that did not escape varied significantly among SAC, with mortality significantly low at 25% (lamdacyhalothrin) and 50% (deltamethrin) SAC (Table 4).

### Field evaluations

#### Thailand

In comparison to the matched control hut, there was a 42.3% and 46.4% increase in the percentage of marked *Ae. aegypti* females that exited huts containing fabric panels treated at FAR and ½FAR alphacypermethrin, respectively (Table 5); although these results were statistically comparable among treatment and control conditions (P = 0.4). In both treatment huts, the percentage of KD mosquitoes collected indoors were comparable but both significantly higher compared to the control hut (P<0.01). Similar results were obtained from analyses of mosquitoes collected inside the exit traps that were dead 24 hours post-escape (P = 0.02) (Table 5). In both treatment huts, more mosquitoes exited during the early sampling periods of 06.00–14.00 Hrs (10.0%±3.8 and 9.7%±1.2 for FAR and ½FAR, respectively) as compared to the untreated control hut (1.9%±1.2) (P = 0.05), which had highest escape later in the day (15.00–18.00 Hrs) (Table S2). Mean indoor temperature (P = 0.9) and relative humidity (P = 0.6) readings were comparable among all huts during the trials (Table 5).

**Table 5:** Percentage escape, indoor KD, escapees mortality of *Ae. aegypti* 1 using alphacypermethrin treated huts in Thailand.

Concentrations (nmoles/cm^2^)	SAC^2^ (%)	Total Nb. Released	Mean (SE) daily escape^3^	Difference % escape treatment and control	Mean(SE) KD (Indoor)	Mean (SE) 24 hour Mort. (escapees)	Mean Temp. (SE) (indoor)	Mean RH (SE) (indoor)
Untreated	75	370	9.70 (2.3)^a^	-	1.80 (1.1)^b^	2.50 (2.5)^b^	24.3 (0.6)^a^	70.4 (2.4)^a^
FAR^4^ (7.2)	75	265	16.8 (6.5)^a^	42.3	29.2 (6.3)^a^	51.4 (11.3)^a^	24.4 (0.6)^a^	70.7 (2.5)^a^
½FAR (3.6)	75	358	18.1 (6.6)^a^	46.4	33.0 (5.9)^a^	29.3 (11.1)^ab^	24.3 (0.6)^a^	67.7 (2.4)^a^

1 Three-seven-day old females, non-blood-fed, 24 hour sugar starved (THAI).

2 Surface area coverage of treated material.

3 For each trial (n = 4 replicates), percent escaping after correcting for knockdown inside the same hut. Means with same letter in superscript within the same column are not significantly different based on one-way ANOVA and Student Newman Keuls (SNK) tests.

4 WHO recommended field application rate (FAR) = 7.2 nm/cm^2^ or 0.03 g/m^2^.

#### Peru

The percentage of *Ae. aegypti* that exited from huts treated with alphacypermethrin at FAR (at all SAC) (P = 0.8) and percentage dead 24 hours post-escape (P = 0.3) was comparable to control, with 0–11% increase in escape compared to the untreated hut (Table 6). However, the percentage of KD mosquitoes inside the huts (P<0.01) and subsequent 24 hour mortality in the same KD populations (P<0.01) were significantly higher in all the huts treated with chemical (even 25% SAC) than in the untreated hut indicating a chemical effect (Table 6). Similar results in escape (P = 0.2) and mortality (P = 0.9) rates 24 hours post-escape were observed using alphacypermethrin at ½FAR treatment (Table 6). Unlike the FAR trials, percentage KD inside the huts (P = 0.2) and 24 hour mortality rates (P = 0.4) in these KD populations were comparable between treated and untreated huts at all SAC (Table 6). The percentage of exiting mosquitoes during each time period was comparable among treated and the untreated hut for each chemical concentration evaluated (Table S3). Mean indoor daily temperature was comparable among huts during both FAR and ½FAR trials (P = 0.6 and P = 0.2, respectively), while RH significantly varied among huts during both experimental trials (P<0.01) (Table 6).

**Table 6:** Percentage escape, indoor KD, and mortality of *Ae. aegypti* 1 using alphacypermethrin treated huts in Peru.

Concentrations (nmoles/cm^2^)	SAC^2^ (%)	Total Nb. Released	Mean (SE) daily escape^3^	Difference % escape treatment and control	Mean (SE) KD (Indoor)	Mean (SE) 24 hour Mort. (indoor)	Mean (SE) 24 hour Mort. (Responders)	Mean Temp. (SE) (indoor)	Mean RH (SE) (indoor)
FAR^4^ (7.2)	100 D untreated	472	80.6 (8.8)^a^	-	1.5 (0.5)^b^	1.5 (0.5)^b^	5.5 (1.4)^a^	24.7 (0.5)^a^	81.0 (0.9)^b^
	100	414	80.6 (8.5)^a^	0	37.1 (7.4)^a^	20.6 (4.2)^a^	4.2 (1.8)^a^	24.1 (0.4)^a^	85.3 (0.8)^a^
	75	452	87.6 (5.3)^a^	8.7	28.7 (12.0)^a^	14.6 (3.4)^a^	7.8 (1.4)^a^	24. 8 (0.5)^a^	80.1 (1.0)^b^
	50	471	82.5 (15.8)^a^	2.4	24.1 (5.5)^a^	12.9 (3.0)^a^	4.5 (1.3)^a^	24.1 (0.4)^a^	84.7 (0.7)^a^
	25	473	89.8 (9.6)^a^	11.4	16.6 (6.1)^a^	9.8 (4.5)^ab^	2.9 (1.4)^a^	23.9 (0.4)^a^	86.1 0.8)^a^
½FAR (3.6)	100 D untreated	456	90.9 (2.8)^a^	-	2.7 (0.6)^a^	2.7 (0.6)^a^	13.2 (4.2)^a^	26.5 (0.3)^a^	80.2 (1.0)^a^
	100	464	81.3 (4.7)^a^	−11.8	6.4 (2.0)^a^	5.8 (2.0)^a^	11.2 (4.1)^a^	27.2 (0.4)^a^	74.5 (1.2)^b^
	75	478	88.9 (2.3)^a^	−2.24	3.4 (1.4)^a^	3.4 (1.4)^a^	11.3 (3.5)^a^	26.0 (0.3)^a^	80.0 (0.9)^a^
	50	487	83.0 (5.6)^a^	−9.5	4.6 (2.1)^a^	4.6 (2.1)^a^	14.4 (5.3)^a^	27.2 (0.4)^a^	74.5 (1.2)^b^
	25	490	91.3 (2.8)^a^	0.4	1.2 (0.6)^a^	1.2 (0.6)^a^	9.2 (4.7)^a^	26.0 (0.3)^a^	81.5 (1.0)^a^

1 Three-seven-day old females, non-blood-fed, 24 hour sugar starved (PERU).

2 Surface area coverage of treated material.

3 For each trial (n = 5 replicates), percent escaping after correcting for knockdown inside the same hut. Means with same letter in superscript within the same column are not significantly different based on one-way ANOVA and Student Newman Keuls (SNK) tests for each treatment concentration.

4 *WHO recommended field application rate (FAR) = 7.2 nm/cm^2^ or 0.03 g/m^2^.*

## Discussion

The objective of the current study was to evaluate the efficacy of contact irritant chemicals to elicit *Ae. aegypti* escape responses from a treated space under both laboratory and field conditions. The approach included treating at concentrations below standards for insect toxicity and at surface area coverage below 100%. The overall goal was to describe the potential of focal application of irritant compounds to drive sublethal vector behaviour (such as escape) that could disrupt human-vector contact. If evident, the role for these approaches in novel control strategies would be strengthened.

In the laboratory, significant escape responses were observed at treatment concentrations equal to but also below corresponding WHO recommended field application rates for the test compounds based on chemical levels required for vector mortality [34]. Our field studies in Thailand corroborate this finding with a 46% increase in the percentage of *Ae. aegypti* exiting a hut treated with alphacypermethrin at ½FAR and significantly more mosquitoes exiting the treated hut prematurely, by four hours, compared to the control hut. The time of vector escape from inside a treated space is as important as the total density of escape since the faster irritation and escape occurs, the greater the probability that contact (i.e., biting) of the human host has or can been prevented. Combined, these results are similar with previous studies conducted in Thailand that found significant escape response in an *Ae. aegypti* local strain to alphacypermethrin tested at concentrations below the WHO FAR and continue to support the fact that insecticides can be used to elicit irritant actions even if applied at concentrations below toxic levels [26], [28], [39].

The current study also evaluated the escape behavior of *Ae. aegypti* in response to different treatment surface area coverage (SAC) of a space using dark∶light fabric panels, where chemical was applied on dark surfaces only representing focal application. The approach of using contrasting colors and treating black fabric alone was based on exploiting known behavior that *Ae. aegypti* adults prefer to rest in dark, damp locations in households, and are generally attracted to black colors [40]–[42]. The evaluation of varying SAC was founded on the theory that taking advantage of resting characteristics could result in desired outcomes achieved with less than 100% treatment of indoor resting sites. A previous laboratory study [31] reported on the resting patterns of *Ae. aegypti* using different dark: light SAC ratios of sublethal concentrations of various pyrethroid chemicals. Results from these experiments indicated that *Ae. aegypti* preferred to rest on dark versus light-colored surfaces, even at the lowest 25% dark coverage and that when preferred *Ae. aegypti* resting sites (i.e., dark surfaces) were chemical-treated, mosquitoes did not significantly alter their resting behavior to safe-sites (untreated areas). Instead, they became agitated, and took to flight rather than seeking out alternate resting sites. However, in that study, the test populations were not provided the opportunity to escape from the test arena so measurements of exiting were not captured [31].

We report here that exposure of *Ae. aegypti* females to alphacypermethrin using FAR at 25% and 50% SAC resulted in escape responses that were comparable with those reported at 100% SAC. In addition, escape responses greater than controls were also observed using treatment coverage at <100% at the ½FAR sublethal concentration of all test compounds. It is noted that in most trials, escape responses were highest when using treated 100%L SAC. This agrees with previous data [31] that showed less overall *Ae. aegypti* resting on light versus dark fabric material. Higher escape responses on 100%L SAC may be due to the combination effect of light color (reduction of preferred resting sites) and the irritating chemical. Field studies from Thailand also indicated a substantial premature escape and an average of 40% increase in exiting an alphacypermethrin-treated hut using 75% SAC. Combined, these results demonstrate that, irrespective of treatment concentrations, substantial escape responses can be elicited in *Ae. aegypti* test populations at treatment coverage below the traditional 100% SAC used in indoor residual spray campaigns. These findings agree with studies conducted in southern Mexico using selective spraying to target the preferred resting sites of *An. albimanus* which showed that reduced coverage application was as effective as full spraying in controlling adult populations [43]. In addition, preferred resting sites, as determined by landing frequencies, times of resting, preferred surface types and resting heights were not modified by the insecticide applications. In essence, the preferred resting sites of the mosquitoes did not change, even on insecticide-treated surfaces after four successive spray rounds [43].

The use of this approach - sublethal concentration and <100% treatment area coverage - against *Ae. aegypti*, or other vector species, if effective, holds the potential for reducing programmatic costs due to reduced requirements for active ingredient. Arredondo-Jimenez et al (1995) showed that selective spraying of preferred resting sites required 46% less time and cost 67% less than conventional full-spraying. Cost and manpower constraints could be further reduced by integrating the proper active ingredients into consumer-based products (pre-treated material strips, plastic sheeting, tiles, wallpaper, paint, etc.) thereby sharing vector control costs with community members. Initial focus group studies evaluating acceptability of sublethal approaches to vector control have indicated that treated materials are viable options for chemical application inside homes [44]. While all trials in the current study were performed with fresh chemical applications, the pyrethroids evaluated are known to have residual properties [34], [45]–[47], therefore their effectiveness over a long period of time would be anticipated, further supporting a cost-effective approach. In addition, such control strategies may increase sustainability of an intervention through home ownership, and broaden the delivery platforms available for traditional house treatment. This concept recognizes that householders in many developing countries already buy pesticides to control insects in the homes [44], [48], and there are advantages in engaging market forces to promote such products, rather than relying solely on public health appeals.

Experimental hut experiments in Peru showed only an 8.7% increase in escape from huts treated with alphacypermethrin at FAR using 75% SAC as compared to an untreated hut and no increase in escape rate when alphacypermethrin was used at ½FAR, irrespective of the SAC. In addition, the time of exit in Peru trials was comparable between treatments and chemical-free control. These results are in contrast to both laboratory and Thailand field trials that showed substantial increases in escape rates for the same compound and treatment coverage in relation to controls. These differences are most likely due to the naturally high exit rate of *Ae. aegypti* observed during baseline studies in Peru when no chemicals were used (Castro et al. unpublished data), making any impact due to chemical difficult to measure, and the variation in fabric material type used between the two field sites (polyester in Thailand versus cotton in Peru). Differences in material type may lead to differences in chemical uptake, absorption and release. Previous studies [49], [50] showed less mosquito KD and mortality when locally sourced mosquito nets (treated with K-O Tab 1-2-3 ‘dip-it-yourself’ long-lasting formulation) were made from cotton as opposed to polyester. Although cotton retained a higher concentration of insecticide, the majority of the chemical is bound within the cotton fibers rather than remaining on the surface where the mosquitoes make contact [49]. However, the chemical was shown to be available for uptake and effect based on indoor KD rates observed within alphacypermethrin-treated huts. The difference between our laboratory findings, where minimal escape occurred from control chambers, and results from our field studies in Peru, where high escape occurred from chemical-free huts, most likely reflects influences from environmental parameters such as temperature and humidity in the micro-environment of the experimental huts. A previous study in Thailand demonstrated how *Ae. aegypti* exit behavior is affected by ambient environmental factors of temperature and humidity [51]. The possibility that behavioral variation between the Thailand and Peru study sites in the current study is the result of the geographically different strains is unknown. These results illustrate that despite considerable progress in the field of vector behavior as a whole, there remains much to understand on how external and genetic factors affect the biology and behavior of important disease vectors.

Both laboratory and field results from the current study showed that KD and mortality were considerably low (<90%) in our *Ae. aegypti* test populations despite using FARs that are based on concentrations required to produce lethal outcomes (i.e. LD90) [52]. *Aedes aegypti* THAI strain from Pu Tuey has been characterized as pyrethroid tolerant [27], [33] while the PERU strain from Iquitos, Peru is most likely susceptible to the chemicals evaluated in this study (Vasquez La Torre, personal communication). It was therefore expected that KD and mortality would be low in test populations from Thailand. However, the low mortality rates observed in *Ae. aegypti* from Peru that did not escape in our laboratory tests could be explained by the minimum exposure time compared to standard resistance testing [52], [53] (i.e. 10 min in a single contact irritancy replicate versus standard one hour used in toxicity assays); also by the potential difference in chemical uptake, absorption and release between the cotton material used in this study and bottle and/or filter test methods used in many resistance testing [52], [53].

More importantly, our *Ae. aegypti* assay cohorts from Thailand continued to exhibit a contact irritant response when exposed to test pyrethroids, despite indication of tolerance, thus indicating that a sublethal approach to vector control may be effective in locations where insecticide resistance occurs. It is worth noting that highest mortality was observed at FAR at 25%D in mosquitoes that did not escape. This finding suggests that although they did not escape, the mosquitoes rested on treated material long enough to receive a lethal dose. Combined, results suggests that escape can occur from a treated area at <100% SAC and of those that do not escape, resting on treated surfaces will continue as supported by a previous study [31] and result in mortality. As expected, we also show that KD and mortality were lower in mosquitoes that escaped as compared to those that did not escape. It is logical that killing those vectors that remain in a treated space (i.e. inside a home) and may have contact with humans will impact pathogen transmission. However, the role of escape and survival for disease management seems counterintuitive. We theorize that an escape and survival response could lead to reduced selection pressure for insecticide resistance which would not only potentially interrupt human-vector contact inside the home through contact irritancy but also extend the effective life of the chemical. Those mosquitoes that are irritated and exit have the opportunity to transfer their genetic material to the next generation, thereby potentially selecting for the mechanistic pathway that result in behavior modification – i.e., irritancy. Of course, this is dependent on linking the mode of action for contact irritancy to one that is dependent on up- or down-regulation of particular cascade drivers. Such information is not yet available. The inclusion of an outdoor trap, such as in a push-pull strategy, could serve to remove irritated vectors from the peridomestic environment thereby controlling potential movement to an untreated location and hosts.

Pertinent to the goal of developing a push-pull strategy, results presented here suggest a role for contact irritancy to drive vectors from a space occupied by a human host, and indicate efficacy using minimal chemical concentration (<LD90) and treatment coverage area (<100%). This in spite of the fact that the chemical delivery platform and formulation we evaluated were not formulated (i.e., optimized for chemical residuality). Although encouraging, current results suggest that behaviors observed in one species or strains might not translate into other mosquito species or strains found in different environmental settings. In addition, the inside environment of an experimental hut is much different than that of a local home; therefore future evaluations of contact irritant efficacy must be performed using indigenous houses against natural *Ae. aegypti* populations. Lastly, although contact irritancy may promote escape, it will be critical to measure the level of reduction, if any, in human-vector contact (i.e., biting) to truly understand the impact of sublethal behaviors on pathogen transmission. The irritant response must occur prior to blood meal acquisition. Due to this challenge, we are therefore currently applying a similar concept of sublethal concentrations and focal treatment application to the use of spatial repellent compounds in a push-pull strategy. This is an attempt to exploit the deterrent actions of chemicals to prevent *Ae. aegypti* entry into homes and thereby reduce indoor densities of mosquito vectors available for human-vector contact. The latter, if successful, may provide greater protection from pathogen transmission as compared to contact irritancy as repellency functions to provide a barrier of protection that is not dependent on vector contact with a treated surface [54].

## Supporting Information

Table S1 Percentage knockdown *Ae. aegypti^1^* that escaped and those that did not escape under laboratory conditions.
(DOC)

Table S2 Percentage escape of *Ae. aegypti^1^* against alphacypermethrin (75% SAC) at designated time periods in Thailand.
(DOC)

Table S3 Percentage escape of *Ae. aegypti^1^* against alphacypermethrin at designated time periods in Peru.
(DOC)
