# Supplementary material for: Contact Irritant Responses of Aedes aegypti Using Sublethal Concentration and Focal Application of Pyrethroid Chemicals
Source: PLoS Negl Trop Dis. 2013 Feb 28;7(2):e2074. doi: 10.1371/journal.pntd.0002074 (PMC3585116; doi:10.1371/journal.pntd.0002074)
Supplement: Table S1 — Percentage knockdown Ae. aegypti1 that escaped and those that did not escape under laboratory conditions. (DOC) [file pntd.0002074.s001.doc]

Table S1. Percentage knockdown *Ae. aegypti^1^* that escaped and those that did not escape under laboratory conditions

| Chemicals | SAC^3^ (%) | Mean^2^ ± SE Percentage knockdown | | | | | |
| --- | --- | --- | --- | --- | --- | --- | --- |
|  |  | Escaped | | | Did not Escape | | |
|  |  | ½FAR^4^ | FAR | *P^5^* | ½FAR | FAR | *P* |
| Alphacypermethrin | 25 | NA^7^ | 0 ± 0^a^ | - | 9 ± 3^ab^ | 36 ± 6^a^ | <0.01 |
|  | 50 | 0 | 0 ± 0^a^ | - | 9 ± 2^ab^ | 34 ± 5^a^ | <0.01 |
|  | 75 | 0 | 0 ± 0^a^ | - | 7 ± 4^b^ | 7 ± 2^b^ | 0.94 |
|  | 100D | 0 | 25 ± 25^a^ | 0.66 | 21 ± 3^a^ | 48 ± 5^a^ | <0.01 |
|  | 100L | 0 ± 0 | 16 ± 4^a^ | <0.01 | 16 ± 3^ab^ | 26 ± 5^a^ | 0.10 |
|  | *P^6^* | - | 0.06 |  | 0.01 | <0.01 |  |
| Lambdacyhalothrin | 25 | 0 ± 0 | 0 ± 0 | *-* | 1 ± 1^b^ | 3 ± 2^ab^ | 0.49 |
|  | 50 | 0 ± 0 | 0 ± 0 | *-* | 0 ± 0^b^ | 5 ± 2^ab^ | 0.02 |
|  | 75 | 0 ± 0 | 0 ± 0 | *-* | 2 ± 2^b^ | 3 ± 2^ab^ | 0.48 |
|  | 100D | 0 ± 0 | 0 ± 0 | *-* | 0 ± 0^b^ | 1 ± 1^b^ | 0.38 |
|  | 100L | 0 ± 0 | 0 ± 0 | *-* | 8 ± 0^a^ | 15 ± 5^a^ | 0.15 |
|  | *P* | *-* | *-* | *-* | <0.01 | 0.01 |  |
| Deltamethrin | 25 | 0 ± 0 | 0 ± 0 | *-* | 0 ± 0^a^ | 0 ± 0^a^ | - |
|  | 50 | 0 ± 0 | 0 ± 0 | *-* | 0 ± 0^a^ | 0 ± 0^a^ | - |
|  | 75 | 0 ± 0 | 0 ± 0 | *-* | 0 ± 0^a^ | 0 ± 0^a^ | - |
|  | 100D | 0 ± 0 | 0 ± 0 | *-* | 1 ± 1^a^ | 1± 1^a^ | - |
|  | 100L | 0 ± 0 | 0 ± 0 | *-* | 1 ± 1^a^ | 1 ± 1^a^ | - |
|  | *P* | - | - | - | 0.56 | 0.89 |  |

^1^Four to seven day old females, non-blood-fed, 24 hour sugar starved (PERU).

^2^ For each trial (n=6 replicates), percentage of knockdown is corrected for control using Abbot's formula. Means in the same column followed by the same letter were not significantly different. Multiple comparisons of means were done using Scheffe’s test (α = 0.05).

^3^Surface area coverage (SAC) of treated material

^4^WHO recommended field application rate (FAR) = 7.2 nm/cm^2^ or 0.03g/m^2^ for alphacypermethrin and lambdacyhalothrin; and 4.9 nm/cm^2^ or 0.025g/m^2^ for deltamethrin.

^5^P values are from t-test examining the effect of treatment concentrations on corrected percentage of knockdown at each treatment coverage.

^6^P values are from one-way ANOVA for difference in corrected percentage of knockdown between treatment coverage at each treatment concentration

^7^NA = Not available (no escapee)
