# Supplementary material for: Contact Irritant Responses of Aedes aegypti Using Sublethal Concentration and Focal Application of Pyrethroid Chemicals
Source: PLoS Negl Trop Dis. 2013 Feb 28;7(2):e2074. doi: 10.1371/journal.pntd.0002074 (PMC3585116; doi:10.1371/journal.pntd.0002074)
Supplement: Table S2 — Percentage escape of Ae. aegypti1 against alphacypermethrin (75% SAC) at designated time periods in Thailand. (DOC) [file pntd.0002074.s002.doc]

Table S2. Percentage escape of *Ae. aegypti^1^* against alphacypermethrin (75% SAC) at designated time periods in Thailand.

| Time Period | Mean (SE) Escape (%)^2^ and [Nb. Escaped/Nb. Available] | | |
| --- | --- | --- | --- |
|  | FAR^3^ | ½ FAR | Untreated |
| T1 (06.00-10.00Hrs) | 2.2 (0.9)^aA^[5/237] | 1.5 (1.5)^aB^ [5/327] | 0.3 (0.3)^aB^ [1/368] |
| T2 (11.00-14.00Hrs) | 7.4 (4.3)^aA^ [15/201] | 7.9 (1.8)^aA^ [21/267] | 1.7 (0.9)^aB^ [6/367] |
| T3 (15.00-18.00Hrs) | 6.4 (3.3)^aA^ [12/184] | 7.8 (1.7)^aA^ [18/237] | 8.6 (2.5)^aA^ [32/363] |

^1^Three to seven day-old females, non-blood-fed, 24 hour sugar starved (THAI).

^2^For each trial (n=4 replicates), percent escaping after correcting for knockdown inside the hut. Means in the same row followed by the same lowercase letter and means in the same column followed by the same uppercase letter were not significantly different based on one-way ANOVA and Student Newman Keuls (SNK) tests.

^3^WHO recommended field application rate (FAR) = 7.2 nm/cm^2^ or 0.03g/m^2^.
