# Supplementary material for: Contact Irritant Responses of Aedes aegypti Using Sublethal Concentration and Focal Application of Pyrethroid Chemicals
Source: PLoS Negl Trop Dis. 2013 Feb 28;7(2):e2074. doi: 10.1371/journal.pntd.0002074 (PMC3585116; doi:10.1371/journal.pntd.0002074)
Supplement: Table S3 — Percentage escape of Ae. aegypti1 against alphacypermethrin at designated time periods in Peru. (DOC) [file pntd.0002074.s003.doc]

Table S3**.** Percentage escape of *Ae. aegypti^1^* against alphacypermethrin at designated time periods in Peru.

| Concentration | Time Period | Mean (SE) Escape (%)^2^ by SAC^3^ | | | | |
| --- | --- | --- | --- | --- | --- | --- |
|  |  | 100D untreated | 100D | 75 | 50 | 25 |
| FAR^4^ | T1 (06.00-10.00Hrs) | 51.4 (8.7)^aA^ | 32.0 (9.6)^aA^ | 45.8 (10.0)^aA^ | 43.8 (6.7)^aA^ | 49.5 (10.8)^aA^ |
|  | T2 (11.00-14.00Hrs) | 16.6 (3.3)^aB^ | 26.0 (3.6)^aA^ | 23.7 (2.1)^aA^ | 20.5 (1.1)^aA^ | 23.3 (4.4)^aAB^ |
|  | T3 (15.00-18.00Hrs) | 11.6 (2.5)^aB^ | 7.4 (4.3)^aB^ | 5.6 (1.9)^aB^ | 6.9 (1.6)^aB^ | 8.9 (4.5)^aB^ |
| ½ FAR (3.6) | T1 (06.00-10.00Hrs) | 40.6 (9.3)^aA^ | 37.7 (6.4)^aA^ | 42.9 (6.1)^aA^ | 37.3 (5.9)^aA^ | 41.8 (7.9)^aA^ |
|  | T2 (11.00-14.00Hrs) | 27.6 (8.8)^aA^ | 22.8 (2.7)^aA^ | 26.4 (3.2)^aAB^ | 28.7 (2.8)^aAB^ | 37.7 (4.4)^aA^ |
|  | T3 (15.00-18.00Hrs) | 20.0 (5.0)^aA^ | 19.9 (6.0)^aA^ | 18.0 (5.6)^aB^ | 15.7 (3.4)^aB^ | 6.5 (2.9)^aB^ |

^1^Three to seven day-old females, non-blood-fed, 24 hour sugar starved Peru strain

^2^For each trial (n=5 replicates), percent escaping after correcting for knockdown inside the hut. Means in the same row followed by the same lowercase letter and the same column followed by the same uppercase letter were not significantly different based on one-way ANOVA and Student Newman Keuls (SNK) tests.

^3^Surface area coverage (SAC) of treated material

^4^WHO recommended field application rate (FAR) = 7.2 nm/cm^2^ or 0.03g/m^2^.
